# Lipoprotein(a) in patients with aortic stenosis: Insights from cardiovascular magnetic resonance

**DOI:** 10.1371/journal.pone.0181077

**Published:** 2017-07-13

**Authors:** Vassilios S. Vassiliou, Paul D. Flynn, Claire E. Raphael, Simon Newsome, Tina Khan, Aamir Ali, Brian Halliday, Annina Studer Bruengger, Tamir Malley, Pranev Sharma, Subothini Selvendran, Nikhil Aggarwal, Anita Sri, Helen Berry, Jackie Donovan, Willis Lam, Dominique Auger, Stuart A. Cook, Dudley J. Pennell, Sanjay K. Prasad

**Affiliations:** 1 CMR Unit, Royal Brompton Hospital and NIHR Biomedical Research Unit, Royal Brompton and Harefield Hospitals and Imperial College London, London, United Kingdom; 2 Norwich Medical School, University of East Anglia, Bob Champion Research & Education Building, Norwich, United Kingdom; 3 The Lipid Clinic, Addenbrooke’s Hospital, Cambridge University Foundation NHS Trust, UK and University of Cambridge, Cambridge, United Kingdom; 4 Department of Statistics, London School of Hygiene and Tropical Medicine, London, United Kingdom; 5 Clinic of Cardiology, Stadtspital Triemli, Zurich, Switzerland; 6 Department of Biochemistry, Royal Brompton and Harefield NHS Trust, London, United Kingdom; 7 Duke National University Hospital, Singapore, Singapore; 8 Cardiovascular Magnetic Resonance Imaging and Genetics, MRC London Institute of Medical Sciences, Imperial College London, Hammersmith Hospital Campus, London, United Kingdom; Nagoya University, JAPAN

## Abstract

**Background:**

Aortic stenosis is the most common age-related valvular pathology. Patients with aortic stenosis and myocardial fibrosis have worse outcome but the underlying mechanism is unclear. Lipoprotein(a) is associated with adverse cardiovascular risk and is elevated in patients with aortic stenosis. Although mechanistic pathways could link Lipoprotein(a) with myocardial fibrosis, whether the two are related has not been previously explored. In this study, we investigated whether elevated Lipoprotein(a) was associated with the presence of myocardial replacement fibrosis.

**Methods:**

A total of 110 patients with mild, moderate and severe aortic stenosis were assessed by late gadolinium enhancement (LGE) cardiovascular magnetic resonance to identify fibrosis. Mann Whitney U tests were used to assess for evidence of an association between Lp(a) and the presence or absence of myocardial fibrosis and aortic stenosis severity and compared to controls. Univariable and multivariable linear regression analysis were undertaken to identify possible predictors of Lp(a).

**Results:**

Thirty-six patients (32.7%) had no LGE enhancement, 38 (34.6%) had midwall enhancement suggestive of midwall fibrosis and 36 (32.7%) patients had subendocardial myocardial fibrosis, typical of infarction. The aortic stenosis patients had higher Lp(a) values than controls, however, there was no significant difference between the Lp(a) level in mild, moderate or severe aortic stenosis. No association was observed between midwall or infarction pattern fibrosis and Lipoprotein(a), in the mild/moderate stenosis (p = 0.91) or severe stenosis patients (p = 0.42).

**Conclusion:**

There is no evidence to suggest that higher Lipoprotein(a) leads to increased myocardial midwall or infarction pattern fibrosis in patients with aortic stenosis.

## Introduction

Aortic stenosis is the most common age-related valvular pathology. Symptomatic patients with severe aortic stenosis have a poor prognosis and there is a need for identification of markers that are mechanistically associated with disease progression. Recently, there has been much interest in the role of Lipoprotein(a) [Lp(a)], a lipoprotein subclass first detected by Berg in 1963,[1] whose physiological function still remains elusive.[2] Lp(a) consists of a cholesterol-rich LDL particle with one molecule of apolipoprotein B100 and an additional protein, apolipoprotein(a), attached via a disulphide bond.[3–5] Increased levels of Lp(a) have been associated with increased risk of calcification of the aortic valve, leading to aortic stenosis.[6,7] Lp(a) has further been associated with an increase in the rate of progression of aortic stenosis, and need for intervention to relieve the pressure overload.[8]

Various mechanisms have been proposed as an explanation for the association between Lp(a) and aortic valve calcification and stenosis. One possible mechanism suggests that after transfer from the bloodstream into the wall of the aortic valve cusps, Lp(a) leads to cholesterol deposition in a manner similar to LDL cholesterol. This is supported by the similarity of the structure of Lp(a) to LDL, particularly as Lp(a) consists of a low-density LDL cholesterol-rich particle bound covalently to apolipoprotein(a), leading to thickening of the aortic valve cusps.[3] Another possible mechanism relates to Lp(a) promoting thrombosis by competing with plasminogen and preventing plasmin from dissolving fibrous clots. This could lead to fibrin deposition and aortic valve calcification.[9] A further mechanism suggests that Lp(a) may bind to fibrin and deliver cholesterol to sites of tissue injury, thus promoting calcification in patients with mild aortic stenosis.[10,11] In addition, it has recently been proposed that autotaxin derived from Lp(a) could promote inflammation and mineralisation promoting valve stenosis.[12]

Aortic stenosis is not merely a pathology of the valve, but affects the left ventricular myocardium as well.[13–16] In a recent study only 35% of patients with moderate or severe aortic stenosis had normal myocardium when assessed by cardiovascular magnetic resonance (CMR), whilst 38% had evidence of midwall myocardial fibrosis and 28% had evidence of subendocardial infarction pattern fibrosis. Myocardial fibrosis, both midwall and infarction pattern, is a strong predictor of adverse outcome in AS. [17] Although it is uncertain by which mechanism Lp(a) promotes aortic calcification and stenosis,[10] if an association of Lp(a) with myocardial fibrosis were to be shown this could have clinical implications as patients with fibrosis have worse outcome.[17,18] Furthermore this could provide an explanation why some patients develop fibrosis whilst others with the same degree of valve stenosis do not, and allow us to better risk-stratify patients from the outpatient setting.

As Lp(a) can affect multiple pathways at a cellular level it is uncertain what contribution, if any, it might have in the development of myocardial fibrosis. On one hand Lp(a) can compete with plasminogen for binding to lysine residues on the surface of fibrin, leading to a reduction of plasmin generation[19] and associated fibrinolysis. This impairment of clot lysis can then lead to increased accumulation of cholesterol[20] and (micro) thrombosis thus increasing the risk of myocardial fibrosis. On the other hand, Lp(a) has been shown to decrease the level of transforming growth factor beta (TGF- β)[21]; a factor promoting myocardial fibrosis in aortic stenosis[15] and other conditions[22] therefore leading to a reduced risk of fibrosis.

The potential association of Lp(a) with myocardial fibrosis in patients with aortic stenosis has not been previously studied. In this study we investigated whether myocardial fibrosis was associated with higher levels of Lp(a) and compared the Lp(a) values in the mild/moderate and severe aortic stenosis groups.

## Methods

Between 2011–2013, consecutive patients with aortic stenosis who underwent CMR with late gadolinium enhancement (LGE) were prospectively included in this sub-study of CMR use in cardiomyopathy (ClinicalTrials.gov Identifier: NCT00930735). The degree of severity of aortic stenosis was defined according to American College of Cardiology/American Heart Association criteria.[23] Patients with clinical suspicion or evidence of current infection or acute coronary syndrome were excluded. Volunteer controls were recruited following local advertising and also underwent CMR. The study was approved by the Royal Brompton Hospital Institutional Review Board and NHS England Research Committee, and undertaken in accordance with the ethical standards of the Declaration of Helsinki. All patients and volunteers provided a signed consent form. Blood tests were collected on the same day as the CMR and analysed as one batch in a biochemistry approved laboratory.

In our institution, CMR is recommended routinely for all patients with severe aortic stenosis and where the clinical team requires further information regarding the severity of aortic stenosis or left ventricular function or aortic dimensions. We excluded patients with disseminated malignancy, severe aortic regurgitation, moderate or severe mitral regurgitation/stenosis, patients with previous valve replacement operations, patients with contraindications to CMR (including pacemaker and defibrillator implantation) and estimated glomerular filtration rate (Cockcroft-Gault equation) of <30 ml/min.

### Data collection

Demographic characteristics and medical history were collected from the patient as well as their hospital records or community records on the day of the CMR. All medical conditions and prescribed medication were recorded. The presence of coronary artery disease was defined as prior coronary revascularization or the presence of significant coronary artery stenosis as assessed by invasive or computed tomography coronary angiography by >50% lumen diameter narrowing of a vessel of 2mm diameter or greater.

### Cardiovascular magnetic resonance

CMR was performed using a 1.5T scanner (Magnetom Sonata or Avanto, Siemens, Erlangen, Germany) and a standardized protocol. The patients were scanned in a supine position with an anterior coil placed over the heart and advanced into the magnet. Initial localiser images were acquired in the transaxial plane with half-Fourier acquisition single short turbo spin echo (HASTE) and free breathing. These images were then utilised to guide acquisition of a vertical long axis (VLA) cine with balanced steady state free precession (SSFP) with breathholding preferably at end expiration- as this is more reproducible. Breathhold SSFP cines in the 2,3 and 4 chamber views were then taken using the short axis scout and VLA images. Four- chamber and 2-chamber cine images at end diastole were then used to plan a stack of short-axis SSFP cine images, from the level of the AV groove and perpendicular to the left ventricular long axis. Subsequently, 10mm contiguous short axis slices were acquired (7mm thickness, 3mm gap) from base to apex. Retrospective ECG gating was predominantly utilised for the cine acquisition. However, prospective triggering was used in patients with arrhythmia, e.g. atrial fibrillation. The sequence parameters for the SSFP cines were TE 1.6ms, TR 3.2 ms, in plane pixel size 2.1 x 1.3mm and flip angle 60°. Aortic valve planimetry and LV volume and mass were calculated from SSFP sequences as previously described by our group.[17] In the aortic stenosis patients ten minutes after injection of 0.1 mmol/kg of gadolinium contrast agent (Gadovist, Schering AG, Berlin, Germany) followed by 10ml saline flush to ensure complete delivery, inversion recovery–prepared spoiled gradient echo images were acquired in standard long- and short-axis views to detect areas of LGE as described for aortic stenosis patients previously [17][24]. Inversion times were optimized to null normal myocardium with images repeated in two separate phase-encoding directions to exclude artifact.

### Image analysis

For quantification of LV function, volumes, mass and aortic valve severity assessment a dedicated software was used (*CMR Tools*, www.cmrtools.com, Cardiovascular Imaging Solutions., London, United Kingdom) and for quantification of myocardial fibrosis a separate dedicated software was used (*CVI* ^*42*^, www.circlecvi.com, Circle Cardiovascular Imaging, Calgary, Canada).

In *CMR Tools* the endocardial and epicardial contours were semi-automatically applied in end-diastole and end-systole and the diastolic LV mass was calculated from the total end-diastolic myocardial volume multiplied by the specific density of the myocardium, as previously described [17]. The severity of aortic stenosis was assessed using CMR-derived planimetry of the aortic valve area. This technique has been validated against echocardiographic measures of aortic stenosis severity.[24] The aortic stenosis was graded using the CMR aortic valve area (AVA) as follows: mild, >1.5 to 2.5 cm^2^; moderate, 1.5 to 1.0 cm^2^; and severe, <1.0 cm^2^ in accordance with the American College of Cardiology/American Heart Association guidelines.[23] For the final classification of stenosis severity for our cohort this method was used.

**Fig 1:**
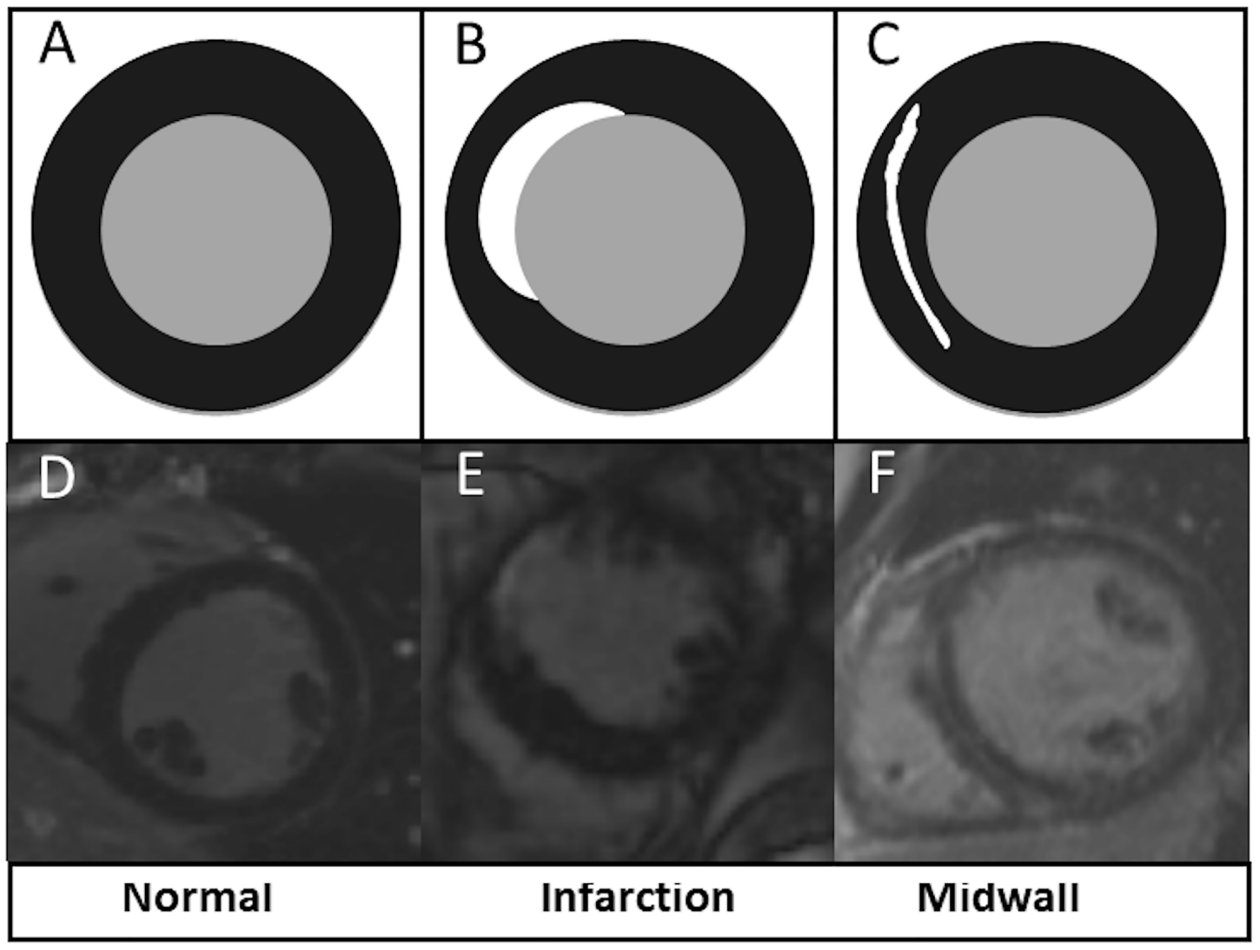
The top panels (A, B, C) represent graphical sketches of a mid-ventricular short axis slice through the myocardium using an inversion recovery sequence. The bottom panels (D, E,F) show the corresponding images obtained with CMR. Panels A and D show normal myocardium with no evidence of fibrosis (homogeneously black following gadolinium administration), panels B and E show infarction pattern fibrosis (subendocardial white enhancement following gadolinium administration) and panels C and F show midwall fibrosis (midwall enhancement following gadolinium administration with normal (black) myocardium both towards the epicardium and endocardium).

**Fig 2:**
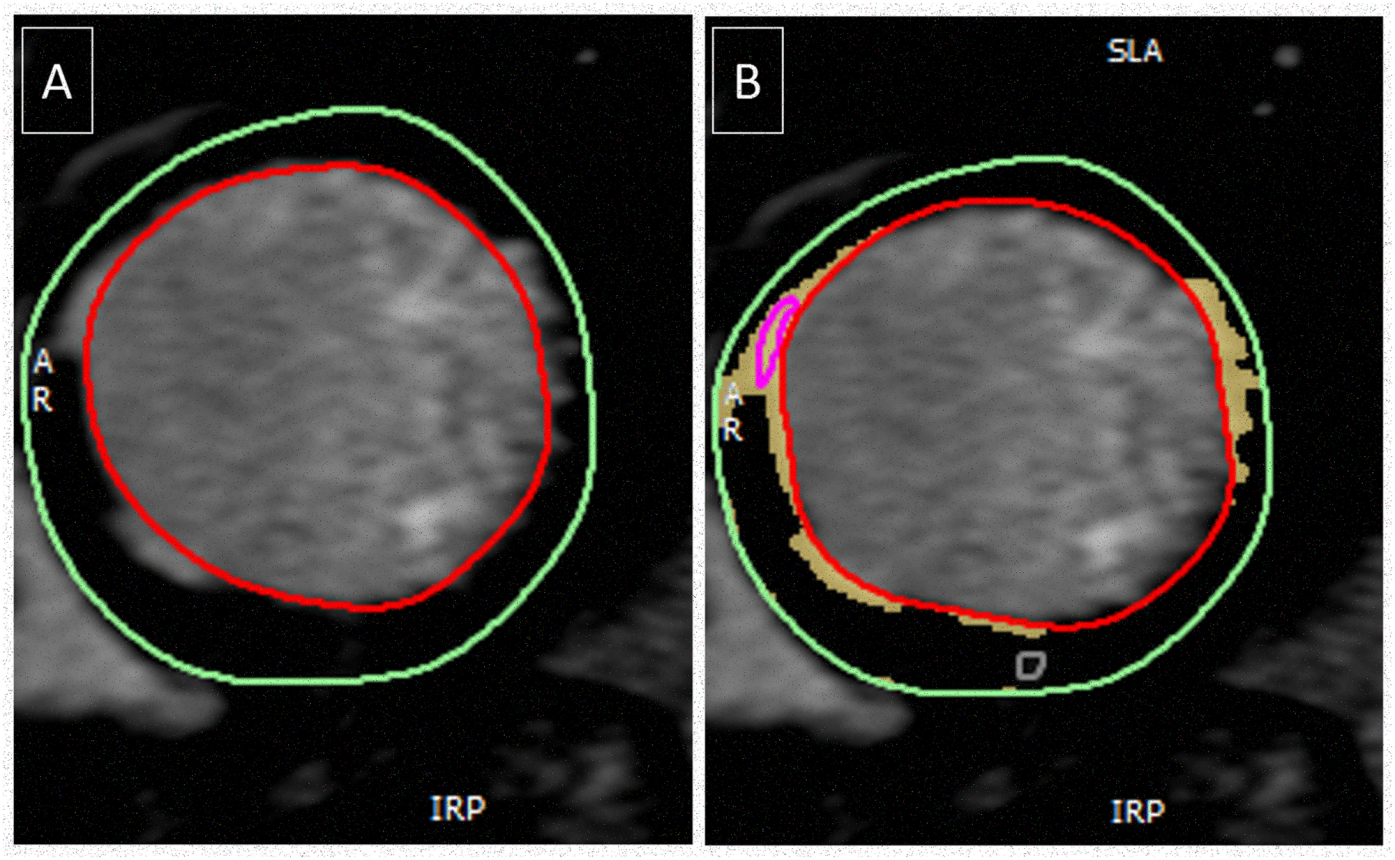
Example demonstrating the quantification of the left ventricular myocardium. Panel A shows the visual late gadolinium enhancement whilst panel B shows the quantified enhanced mass. Once completed for all the myocardial slices then the overall absolute enhanced mass or % mass can be calculated.

The presence and pattern of LGE were assessed by two independent expert observers (SCMR/ EuroCMR Level III) to categorise each patient according to the visual presence or absence of myocardial fibrosis, and if present whether this was midwall fibrosis or infarction pattern fibrosis with examples shown in Fig 1. Both observers were blinded to clinical data. A third blinded observer adjudicated when there was a disparity between the initial two observers. Patients with a mixed pattern of LGE were categorized according to the predominant pattern of fibrosis. The anonymised images of the patients who had fibrosis were then quantified using *CVI* ^*42*^ with the established “full with half maximum” [17] technique and presented as the percentage of enhanced mass in the late phase following gadolinium administration (LGE mass) divided by the total LV mass giving % LGE mass (LGE mass/ total mass) as shown in (Fig 2).

### Lipoprotein(a)

Lp(a) was measured using *Sentinel Diagnostics* Lp(a) Ultra, an isoform independent latex immunoassay developed for Lp(a) levels. When an antigen-antibody reaction occurred between Lp(a) in a sample and anti-Lp(a) antibody, this resulted in agglutination detected as an absorbance change, with the magnitude of the change being proportional to the quantity of Lp(a) contained in the sample. This analysis was undertaken on serum from our patients taken on the day of CMR and stored in a dedicated space in a biobank freezer at -80°C until the day of analysis.

### Statistical analysis

Baseline patient characteristics are presented as mean and standard deviation for continuous variables and number (percentage) for categorical variables. The mild and moderate groups were merged into one group to increase group numbers and directly compared with the severe group. Mann Whitney U tests were used to assess whether there was evidence of an association between Lp(a) and aortic stenosis severity (mild/moderate or severe), and also between Lp(a) and presence or absence of myocardial fibrosis. Finally, univariable and multivariable linear regression analysis were undertaken to identify possible predictors of Lp(a). A p value of <0.05 was taken as significant. All analyses were undertaken using Stata 14.0 (College Station, Texas, USA).

## Results

In total, 110 patients with mild/moderate or severe aortic stenosis were recruited and completed CMR examination and 55 control volunteers. Patient baseline characteristics are shown in Table 1. The baseline pharmacotherapy is shown in Table 2.

**Table 1:** Patient and control demographic characteristics.

**Demographics**	**Mild / Moderate (N = 35)**	**Severe (N = 75)**	**P-Value**
Age, years	71 ± 10	78 ± 9	<0.001
Male, n (%)	26 (74.3)	51 (68.0)	0.66
Hypertension, n (%)	13 (40.6)	44 (58.7)	0.096
Diabetes mellitus, n (%)	1 (3.8)	4 (6.1)	1.000
Any coronary artery disease, n (%)	13 (37.1)	27 (36.0)	1.00
Previous stroke, n (%)	1 (2.9)	2 (2.7)	1.00
Atrial Fibrillation, n (%)	5 (14.3)	6 (8.0)	0.32
Hypercholesterolaemia, n (%)	18 (58.1)	50 (67.6)	0.38
NYHA ≥ II	19 (59.4)	60 (81.1)	0.028
**Demographics**	**All Aortic Stenosis (N = 110)**	**Controls (N = 55)**	**P-Value**
Age, years	76 ± 10	74 ± 7	0.052
Male, n (%)	77 (70.0)	39 (70.9)	1.00
Hypertension, n (%)	57 (53.3)	20 (36.4)	0.047
Diabetes mellitus, n (%)	5 (5.4)	10 (18.2)	0.022
Any coronary artery disease, n (%)	40 (36.4)	26 (47.3)	0.18
Previous stroke, n (%)	3 (2.7)	1 (1.8)	1.00
Atrial Fibrillation, n (%)	11 (10.9)	2 (3.6)	0.14
Hypercholesterolaemia, n (%)	68 (64.8)	27 (49.1)	0.064
NYHA ≥ II	79 (71.8)	6 (11.5)	<0.0001

Top panel comparison between mild/moderate and severe patients with aortic stenosis. Bottom panel comparison between all patients with aortic stenosis and controls. NYHA = New York Heart Association classification.

**Table 2:** Baseline pharmacotherapy of patients and controls at the time of inclusion in the study.

**Medical therapy**	**Mild / Moderate**	**Severe**	**P-Value**
Aspirin, n (%)	19 (61.3)	44 (59.5)	1.00
Clopidogrel, n (%)	4 (13.8)	12 (16.7)	1.00
ACE I/ ARB, n (%)	13 (43.3)	38 (51.4)	0.52
Beta Blocker, n (%)	14 (46.7)	32 (44.4)	1.00
Calcium channel blocker, n (%)	8 (26.7)	6 (8.7)	0.028
Diuretic, n (%)	14 (43.8)	43 (58.1)	0.21
Warfarin, n (%)	4 (14.3)	6 (8.3)	0.46
Amiodarone, n (%)	0 (0.0)	4 (5.6)	0.32
Statin, n (%)	20 (66.7)	54 (72.0)	0.64
**Medical therapy**	**All Aortic Stenosis**	**Controls**	**P-Value**
Aspirin, n (%)	63 (60.0)	26 (47.3)	0.14
Clopidogrel, n (%)	16 (15.8)	25 (45.5)	<0.001
ACE I/ ARB, n (%)	51 (49.0)	24 (43.6)	0.62
Beta Blocker, n (%)	46 (45.1)	20 (36.4)	0.31
Calcium channel blocker, n (%)	14 (14.1)	7 (12.7)	1.00
Diuretic, n (%)	57 (53.8)	3 (5.5)	<0.0001
Warfarin, n (%)	10 (10.0)	0 (0.0)	0.015
Amiodarone, n (%)	4 (4.0)	0 (0.0)	0.30
Statin, n (%)	74 (70.5)	27 (49.1)	0.010

Top panel comparison between patients with mild/ moderate and severe aortic stenosis. Bottom panel comparison between all patients with aortic stenosis and controls. ACE I = Angiotensin converting enzyme inhibitor, ARB = Angiotensin II blocker

### CMR assessment of myocardial fibrosis

Of the cohort, 36 patients (32.7%) did not show any LGE indicating that there was no macroscopic myocardial replacement fibrosis. A total of 38 (34.6%) patients had midwall enhancement suggestive of midwall fibrosis and 36 (32.7%) patients showed evidence of subendocardial myocardial fibrosis, a pattern typical for myocardial infarction. CMR and important biochemical data are shown in Table 3.

**Table 3:** Patient biochemical and CMR characteristics per aortic stenosis severity group.

Biochemical and CMR data	Mild / Moderate	Severe	P-Value
Lp(a), mg/L	420 ± 344	404 ± 390	0.64
Creatinine, μmol/L	93 ± 30	102 ± 36	0.20
CMR aortic valve area, cm^2^	1.2 ± 0.3	0.7 ± 0.1	<0.00001
LVEF, %	62 ± 14	57 ± 17	0.11
LV Mass, g	166 ± 47	168 ± 57	0.87
CMR Myocardial Tissue Characterisation			
No Myocardial Fibrosis, n (%)	13 (37.1)	23 (30.7)	0.82
Midwall Fibrosis, n (%)	11 (31.4)	27 (36.0)	
Infarction Pattern Fibrosis, n(%)	11 (31.4)	25 (33.3)	
Lp(a) by CMR Fibrosis Group			
No Myocardial Fibrosis, mg/L	377 ± 416	418 ± 406	0.93
Midwall Fibrosis, mg/L	421 ± 333	360 ± 389	0.46
Infarction Pattern fibrosis, mg/L	469 ± 278	438 ± 389	0.74

### Lipoprotein(a) level

**Fig 3:**
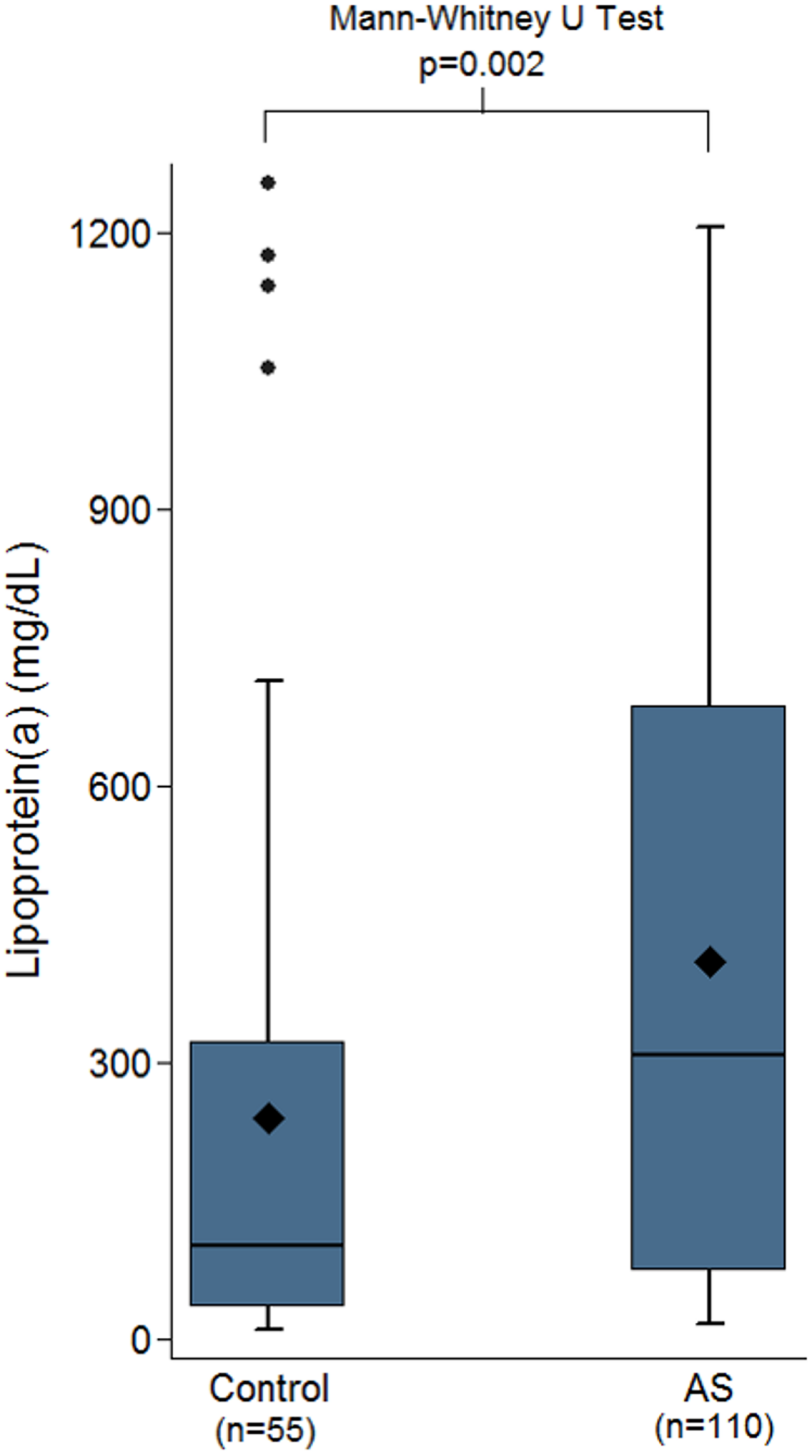
Box plots comparing controls vs. the whole cohort of aortic stenosis patients indicating that the controls had significantly lower Lp(a), median 100mg/L vs 309mg/L, p<0.001.

The controls had a lower median Lp(a) valued compared to the whole cohort of aortic stenosis patients (100 mg/L (41–266) vs 309 mg/L (75–688), p<0.001 as shown in Fig 3).

**Fig 4:**
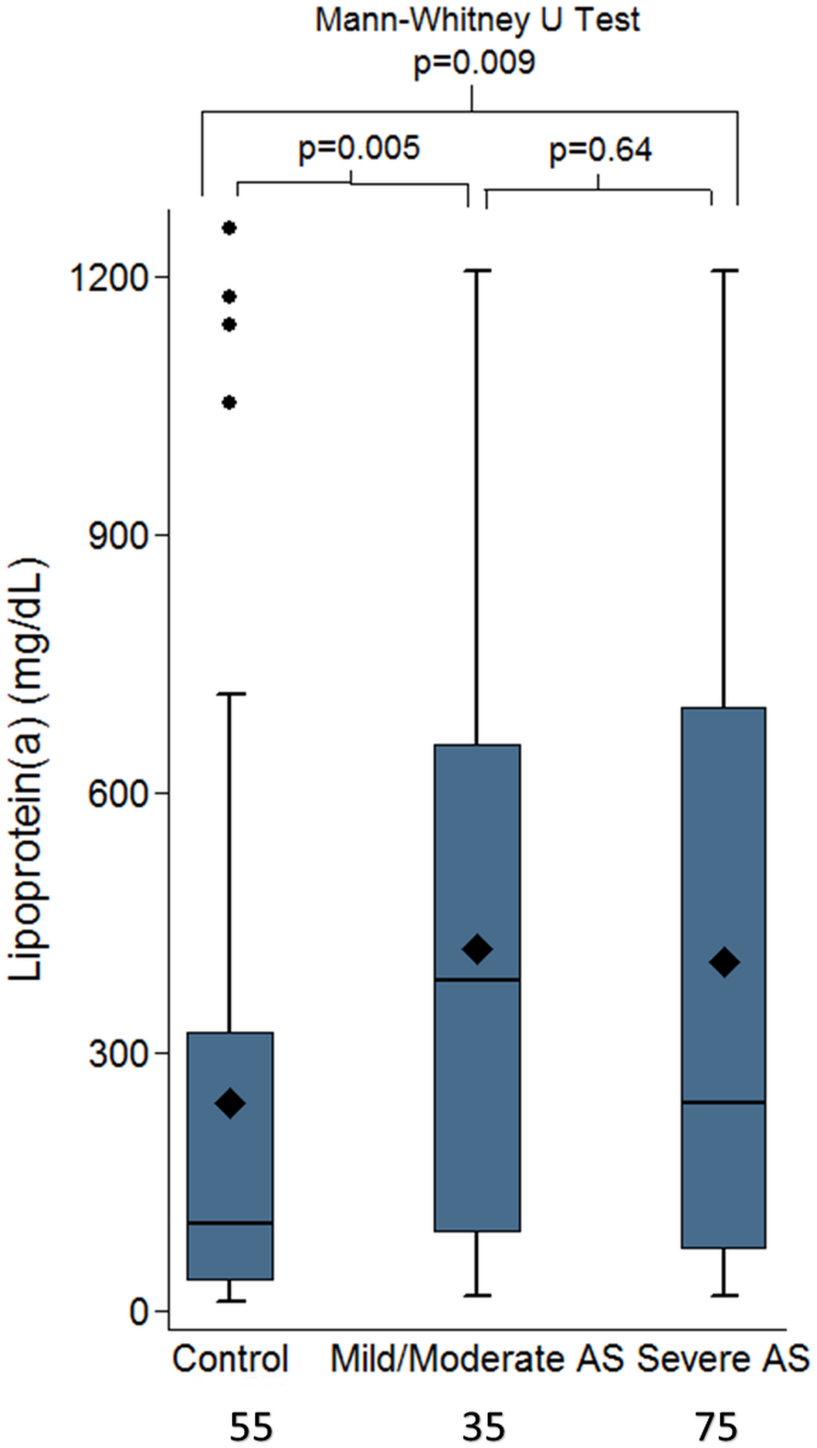
Box plots comparing the controls vs the mild/moderate aortic and severe aortic stenosis patients confirming a significant difference between the controls and either of the groups.

Even when compared to the mild/moderate and severe aortic stenosis group separately there was still a significant difference as shown in Fig 4. Linear regression adjusted for age and sex also confirmed that controls had lower Lp(a) values compared to the patients with mild/moderate (p = 0.013) or severe aortic stenosis (p = 0.019).

**Fig 5:**
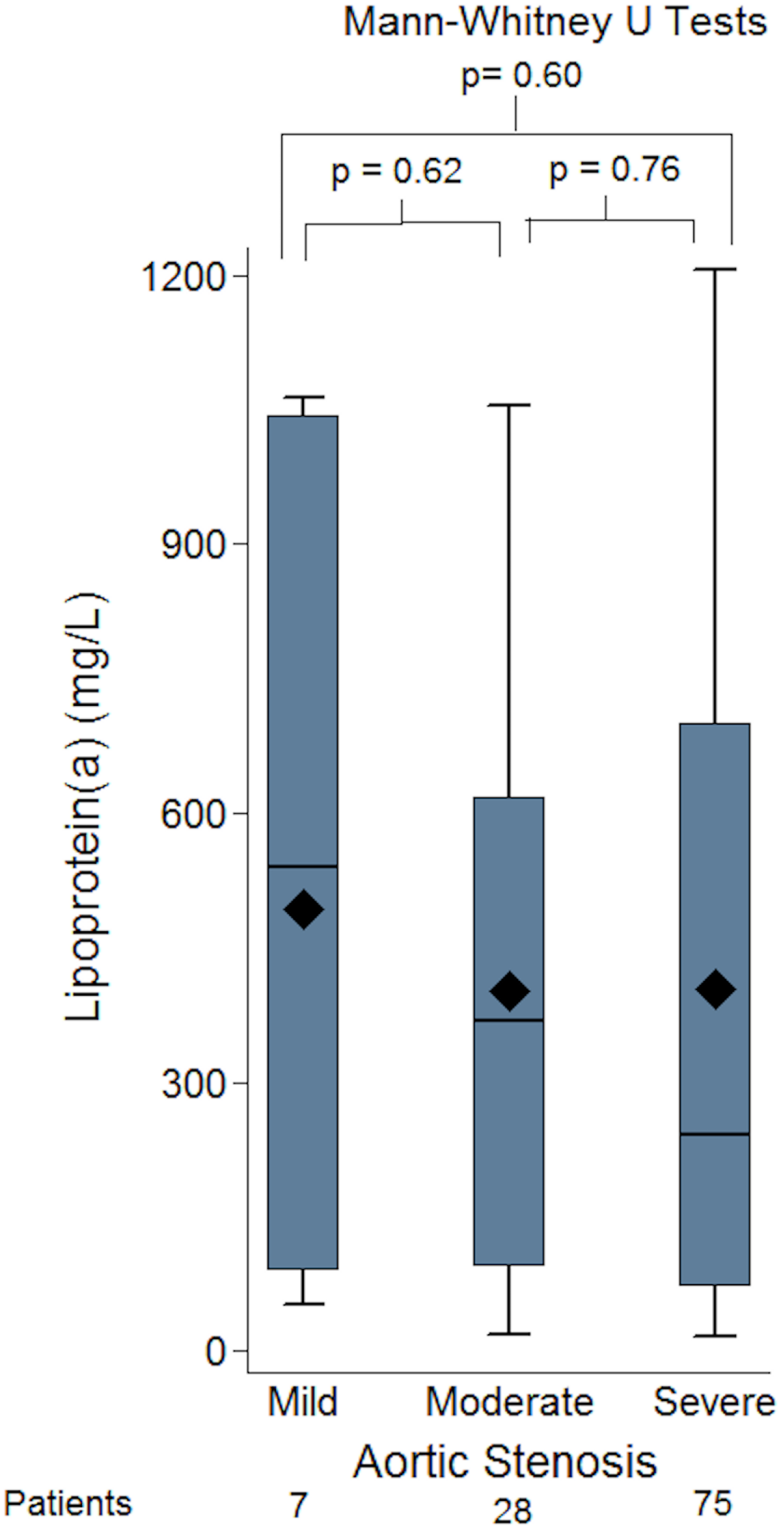
Box-plots of groups of patients with mild, moderate and severe aortic stenosis (AS) and lipoprotein(a) (Lp[a]) level, showing no significant difference between the groups by severity of AS and Lp(a) levels.

Furthermore, there was no significant difference between the Lp(a) level seen in the mild, moderate and severe aortic stenosis group, values 541 (91–1043), 368 (94–619) and 242 (72–700) respectively (Fig 5).

**Fig 6:**
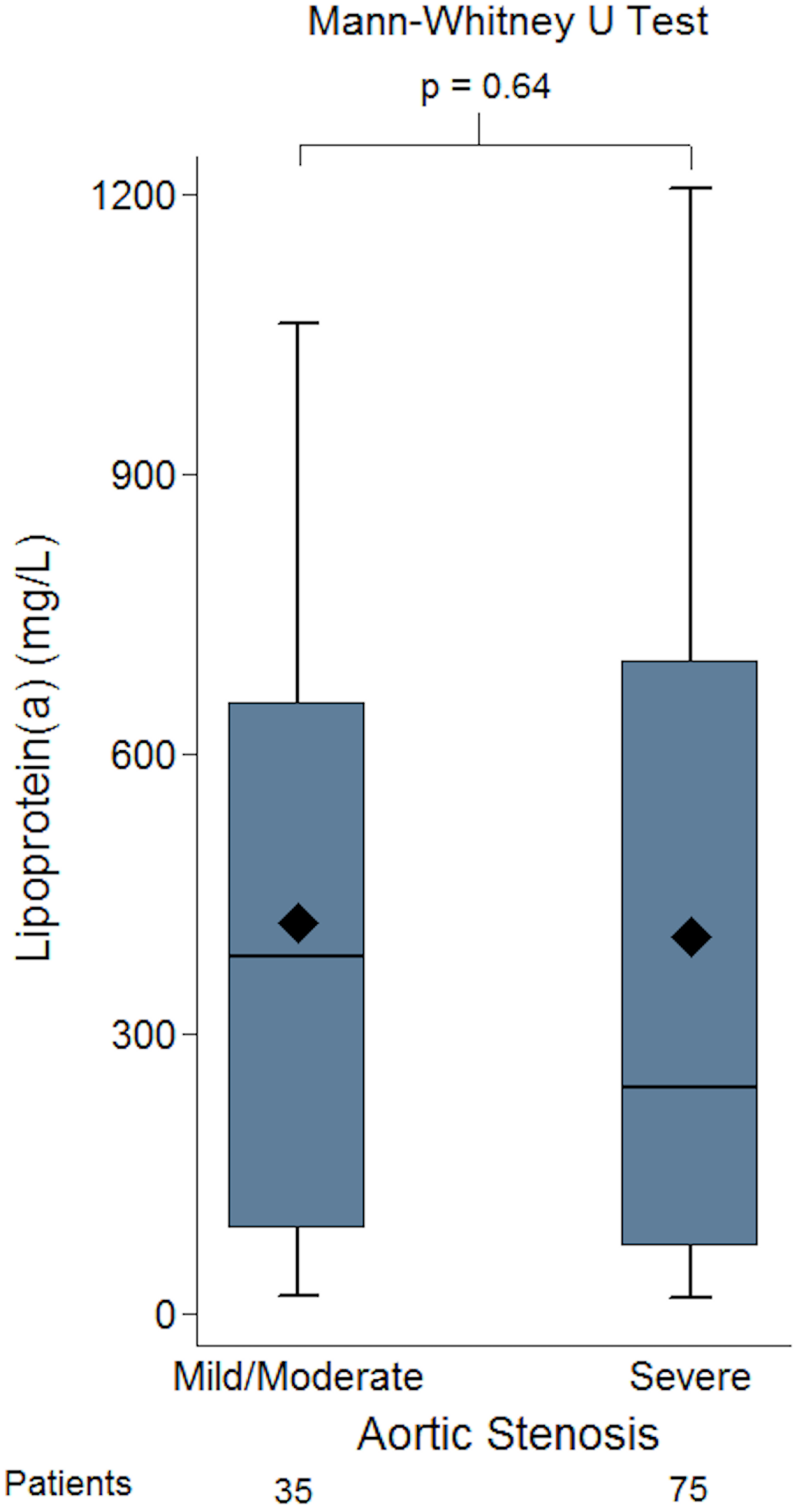
Box-plots of lipoprotein(a) (Lp[a]) concentration in groups of patients with mild/moderate and severe aortic stenosis (AS). There was no difference in the level of Lp(a) in the patients whether they had mild/moderate or severe AS.

The concentration of Lp(a) seen in mild/ moderate aortic stenosis (AVA = 1.0–2.5cm^2^) and severe aortic stenosis (AVA<1.0cm^2^) was compared using the Mann-Whitney U test. The median value for the mild/moderate aortic stenosis group was 384mg/L (91–656) and for the severe group was 242 mg/L (72–700). There was no significant difference between aortic stenosis severity (mild/moderate vs severe) and level of Lp(a), p = 0.64 (Fig 6). Even when the mean Lp(a) values were compared this did not show any statistical difference between the mild/ moderate group and severe aortic stenosis groups (420±344 vs. 404±390, p = 0.84).

Although we did not expect statin use to be a confounder, as statins do not appear to affect Lp(a) especially in non-Familial Hypercholesterolemia populations [25] this was further investigated. Median Lp(a) for the patients not taking statin vs. patients on statins was not different (321mg/L (63–582) vs 324mg/L (97–732), p = 0.25. Moreover, the effect of severity of aortic stenosis on Lp(a) was assessed using multivariable regression including statin use, age, sex, coronary artery disease and presence of fibrosis which failed to show any association (p = 0.78).

**Fig 7:**
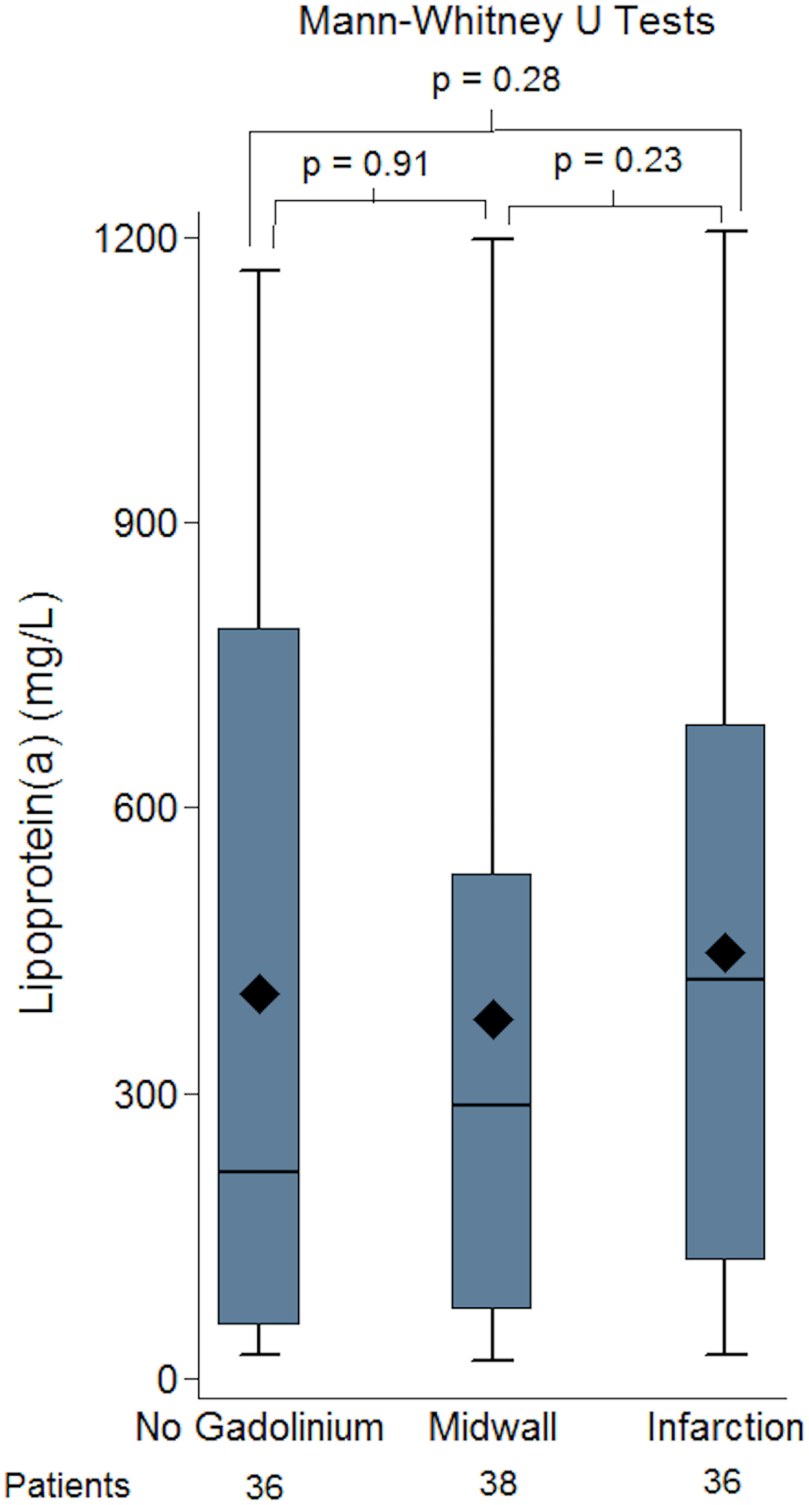
Values in the three groups (no fibrosis/no gadolinium, midwall fibrosis, infarction fibrosis) were compared and no significant difference between the groups was identified. We also assessed whether there was any association between the quantified mass or % enhanced myocardium for either midwall and infarction and Lp(a). There was no association between an increase in the enhanced absolute mass or % enhanced mass (defined by enhanced mass/overall mass) as shown in Fig 8.

**Fig 8:**
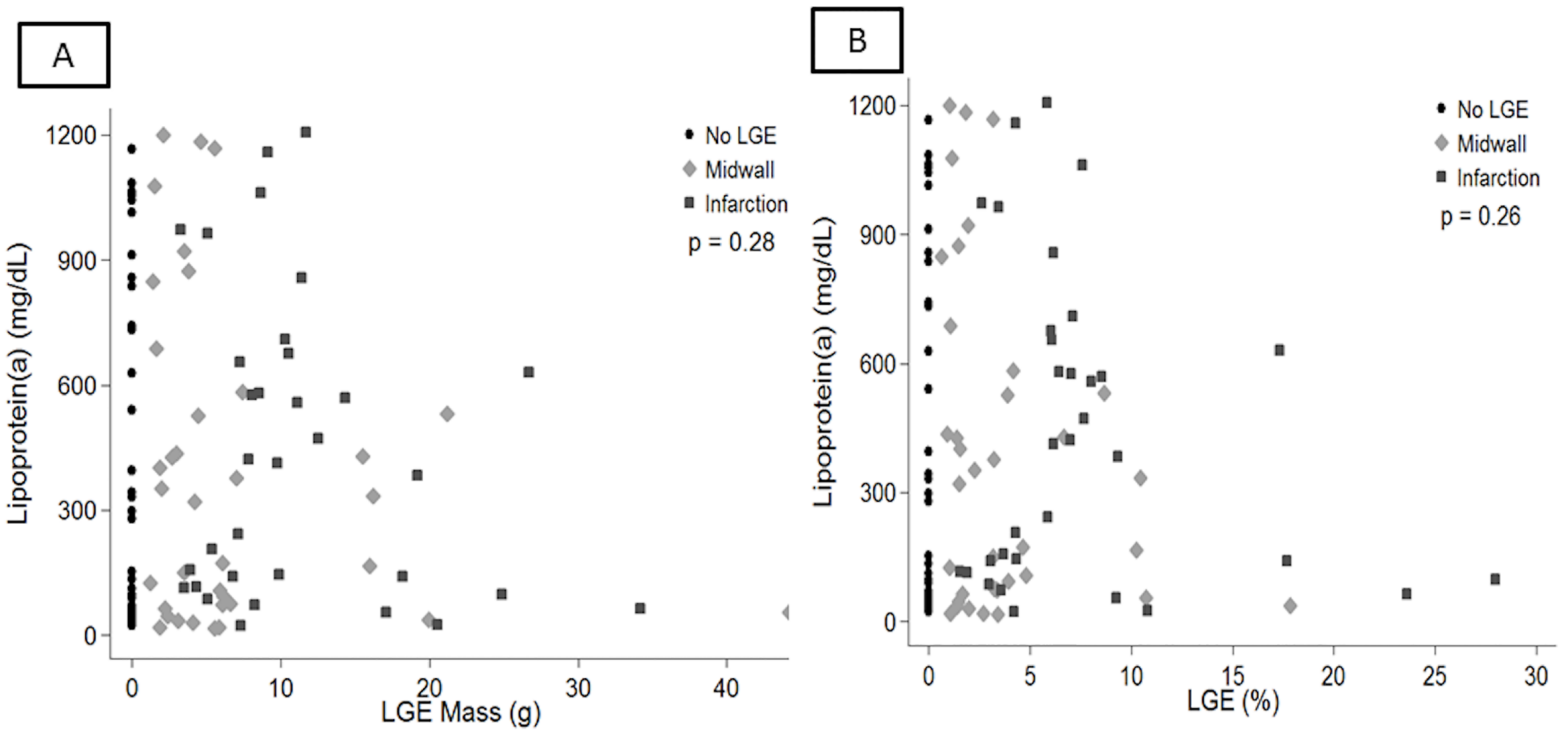
investigating a potential association between quantified enhanced myocardial mass or % mass and Lp(a). As shown in panel A, there was no association between enhanced mass and Lp(a) (p = 0.28). Reviewed separately there was no association between Lp(a) and midwall fibrosis (p = 0.20) or infarction (p = 0.31). Similarly there was no significance for enhanced % mass as shown in panel B.

We further evaluated whether Lp(a) was associated with midwall or infarction pattern fibrosis. As there was no difference between the mild/moderate and severe aortic stenosis groups and Lp(a) level these were merged for subsequent analysis. No association between the presence and absence of fibrosis and Lp(a) was identified (Fig 7). Similarly, there was no association between an increase in the enhanced absolute mass or % enhanced mass (defined by enhanced mass/overall mass) as shown in Fig 8.

We also investigated the prognostic role of Lp(a) in patients developing post-operative LBBB or requiring a pacemaker following surgical aortic valve replacement (AVR) or percutaneous transcatheter aortic valve replacement (TAVI). As shown in Table 4 we found no such association.

**Table 4:** Investigating the potential association between Lp(a) and post-operative new LBBB or need for pacemaker implantation in patients with TAVI or AVR.

	TAVI/AVR			TAVI			AVR		
In those with intervention:	n	Lp(a) [Median (IQR)]	p	n	Lp(a) [Median (IQR)]	p	n	Lp(a) [Median (IQR)]	p
No post-intervention LBBB	49	297 (72–721)	0.92	22	153 (63–558)	0.61	28	412 (86–866)	0.53
Post-Intervention LBBB	10	287 (86–577)		6	362 (35–1077)		4	258 (88–432)	
	TAVI/AVR			TAVI			AVR		
In those with intervention:	n	Lp(a) [Median (IQR)]	p	n	Lp(a) [Median (IQR)]	p	n	Lp(a) [Median (IQR)]	p
No post-intervention PPM	41	297 (86–838)	0.45	17	242 (70–628)	0.65	25	423 (86–858)	0.85
Post-Intervention PPM	15	156 (63–577)		10	151 (35–577)		5	401 (89–427)	
	TAVI/AVR			TAVI			AVR		
In those with intervention:	n	Lp(a) [Median (IQR)]	p	n	Lp(a) [Median (IQR)]	p	n	Lp(a) [Median (IQR)]	p
No post-intervention LBBB/PPM	38	275 (72–838)	0.72	16	196 (58–593)	0.88	23	423 (72–873)	0.71
Post-Intervention LBBB/PPM	18	245 (75–577)		11	156 (35–1063)		7	401 (86–436)	

There was no association between Lp(a) and either post-operative LBBB or need for PPM in TAVI, AVR or the combination of the two.

Furthermore, we evaluated associations between other potential adverse predictors in AS with Lp(a) value. Univariable linear analysis per Lp(a) 100mg/L, was undertaken between patients in the midwall fibrosis vs. no fibrosis, infarction pattern fibrosis vs. no fibrosis and any fibrosis (midwall or infarction) vs. no fibrosis. No association was found between Lp(a) and either fibrosis pattern (midwall p = 0.77; infraction pattern p = 0.62, any fibrosis p = 0.91). There was no correlation between Lp(a) and any other parameters including left ventricular ejection fraction (Spearman correlation 0.14, p = 0.14), left ventricular hypertrophy (Mann-Whitney U Test p = 0.22), left ventricular mass (correlation 0.04, p = 0.68), gender (female median = 577, (IQR 111–741), men 172, (72–558), Mann-Whitney U test p = 0.10); age (Spearman correlation 0.03, p = 0.72); aortic valve area (Spearman correlation 0.09, p = 0.35), evidence of pre-existing coronary artery disease (Mann-Whitney U test p = 0.61) or C-Reactive Protein (Spearman correlation 0.16, p = 0.13).

Our intention in this manuscript was to investigate a potential mechanistic association between Lp(a) and fibrosis. As such as our cohort included patients with moderate and severe aortic stenosis who had medical or interventional therapy. Nonetheless, despite the heterogeneity of patients, it is of interest to review the impact of Lp(a) in outcomes. In our cohort 79 people (71.8%) died or had an aortic valve intervention over a median of 1.9 years (1.2–2.7 years). As Lp(a) has been shown to associate with worse outcome only if very high, we have investigated whether the patients in the highest decile of Lp(a) had worse outcome, defined as overall death or aortic valve intervention, compared to the rest of the cohort. Using Cox proportional modeling we observed a trend towards worse survival or need for intervention in those in the higher decile HR = 1.47 CI 0.76–2.85, p = 0.26, but this did not reach statistical significance.

## Discussion

There has been recent growing interest in the role of Lp(a) in aortic stenosis as Lp(a) has been shown to be causally associated with increased calcification and the need for aortic valve replacement [8,26]. Plasma Lp(a) level is mostly genetically determined by a variation in kringle IV type 2 (KIV-2) repeat numbers at the *LPA* gene, which encodes for apolipoprotein(a) [3]. Recently, genome wide association studies have identified more frequent genetic variations such as the SNP rs104555872 at the *LPA* gene in patients with aortic valve calcification and stenosis and importantly, presence of such genetic variations in the LPA gene led to increased levels of both Lp(a) [27] and clinical aortic stenosis, confirming the causal role of Lp(a) [6]. Despite this breakthrough however, the mechanism by which Lp(a) might promote this remains unclear. Seminal to this, it also remains to be seen whether Lp(a) associates with increased likelihood of myocardial changes as reflected by increased myocardial fibrosis. In addition, clinically what needs to be determined is whether lowering Lp(a) in patients with mild/ moderate aortic stenosis might alter the rate of stenosis progression and subsequent need for intervention. An initial pilot study is currently recruiting to investigate this, the Early Aortic Valve Lipoprotein(a) Lowering Trial (EAVaLL) (ClinicalTrials.gov Identifier: NCT02109614) [28] where patients with aortic sclerosis or mild stenosis are randomized to niacin or placebo. The primary end-point is calcium score progression by cardiac CT in the patients randomized to niacin vs. placebo at two years. Therefore, Lp(a) could provide a novel therapeutic target in addressing this clinically unmet need. At the same time, presence of myocardial fibrosis is an adverse predictor of survival and higher levels of Lp(a) could potentially lead to increased or decreased myocardial fibrosis, depending on the dominant signalling pathway.

This is the first report to explore the potential mechanistic role of Lp(a) in contributing to left ventricular myocardial fibrosis and we observed no evidence to support an association between Lp(a) and ventricular fibrosis. Moreover, in the patients with CMR evidence of myocardial infarction, the Lp(a) level was not significantly different to the other groups, supporting that Lp(a) mediated thrombosis is less likely to be implicated, perhaps as the Lp(a) is not very high. Furthermore, it is also likely that the two opposing mechanisms influenced by Lp(a), one promoting and one reducing fibrosis are running in parallel leading to an overall neutral effect. This finding is reassuring as it suggests that although Lp(a) increases calcification and need for intervention, it is not per se associated with the increased arrythmogenicity and mortality seen in patients with myocardial fibrosis.

Moreover, we observed no association between the level of Lp(a) concentration and the severity of aortic stenosis. Although this was an unexpected finding this lack of association could suggest that Lp(a) might potentially have an initial effect in promoting early calcification; however, once beyond initial stenosis the calcification pathway is independent of Lp(a) hence explaining why in our study we observed no significant difference between mild/moderate and severe.

Finally, although our study was not intended to investigate a potential association of Lp(a) and outcomes in view of the heterogeneity of this cohort, nonetheless, we observed that the patients in the highest decile of Lp(a) levels had an almost 50% higher risk of mortality or need for intervention compared to the patients in the lowest decile, although this did not reach statistical significance.

### Study limitations

A limitation of our study is that it is from a single centre with a high proportion of Caucasian patients. It remains to be shown whether these results could be extended to other races. Secondly, in aortic stenosis the myocardial subendocardial infarction pattern could relate to either atherosclerotic coronary disease or embolic disease, therefore skewing the potential effect of Lp(a). However, even when a history of documented CAD was adjusted for, there was no association of fibrosis with Lp(a) level. It is important to note however, that Lp(a) is only significantly associated with increased risk of myocardial infarction when >500mg/L [3]. As the majority of our patients had lower values this could explain the lack of association. Thirdly, our cohort included 14 patients with bicuspid aortic valve and given that Lp(a) has a genetic component this could have influenced the results. However, even when the 14 patients with bicuspid aortic valve were excluded from the analysis, this did not influence the result outcome. Fourthly, our study of 110 patients is the largest study to date comparing Lp(a) and myocardial fibrosis assessed with CMR. Despite this, there were only 38 patients in the midwall fibrosis group and 36 in the infarction pattern group. It is possible therefore that with higher numbers a small association might have been observed between Lp(a) and fibrosis pattern. We have estimated however, that using the currently available sample size and the standard deviation of Lp(a), at a significance level of 0.05 there would be 80% power to detect a mean difference of 200 mg/L or greater between mild/moderate and severe groups. Combined with the fact that we did observe significant difference between controls and patients with any degree of aortic stenosis this would further support that our study was powered to detect clinically relevant differences in Lp(a).

Likewise, our mild and moderate groups had low numbers which could have stopped us from observing a small difference in Lp(a) level. A final limitation of our study is that we did not have a comparison between Lp(a) and interstitial diffuse fibrosis as quantified by T1 mapping sequences as this might have provided further useful information. When we undertook this study we did not have a validated T1 mapping sequence with appropriate quality assurance to use. It was only subsequently that validated T1 mapping sequences with appropriate quality assurance have been used in aortic stenosis [29][30][31]. Nonetheless, although the strong prognostic role between midwall fibrosis and infarction pattern fibrosis in aortic stenosis has been shown [32], it remains unclear whether T1 mapping could offer incremental benefit, and the association of T1 mapping with Lp(a) remains to be studied.

## Conclusion

In conclusion, our study has shown no association between Lp(a) and left ventricular midwall or infarction pattern fibrosis when compared to patients with no fibrosis, therefore suggesting that Lp(a) is unlikely to mediate fibrosis in patients with aortic stenosis. Additionally, patients with mild/moderate and severe aortic stenosis have similar levels of Lp(a). The mechanistic influence of Lp(a) in patients with aortic stenosis remains uncertain and future studies should aim not only to identify this, but also establish whether a reduction in Lp(a) in the early stages of mild aortic stenosis in the patients with very high Lp(a) levels, either using high dose niacin[5], apheresis[33][34] or novel PKCS9 inhibitors[35] might improve stenosis progression and outcomes.
